# Treatment Patterns and Survival in Older Patients with FIGO Stage IIB–IV Epithelial Ovarian, Fallopian Tube, and Primary Peritoneal Carcinomas

**DOI:** 10.3390/jcm15207845

**Published:** 2026-10-12

**Authors:** Tingting Zhang, Bing Liang, Cuiqin Sang, Zhengchen Guo, Weihua Li, Shuiqing Xu, Hua Li

**Affiliations:** Department of Obstetrics and Gynecology, Beijing Chaoyang Hospital, Capital Medical University, No. 8 Gongti South Road, Chaoyang District, Beijing 100020, China

**Keywords:** older adults, epithelial ovarian cancer, primary debulking surgery, neoadjuvant chemotherapy, progression-free survival, overall survival, retrospective cohort

## Abstract

**Background**: Older adults with epithelial ovarian cancer (EOC) are underrepresented in prospective studies, and treatment selection is influenced by frailty, comorbidity, disease burden, and clinician assessment. We evaluated treatment patterns, operative outcomes, and survival associations in a single-center cohort of patients aged 70 years or older with FIGO stage IIB–IV EOC-spectrum malignancies. **Methods**: We screened 122 patients treated between January 2010 and December 2023 and included 97 patients after exclusions. The primary outcomes were progression-free survival (PFS) and overall survival (OS). PFS excluded seven patients recorded as receiving no treatment, whereas OS included all 97 patients. Kaplan–Meier methods were used to estimate PFS and OS, and overall log-rank tests were used to compare survival across age groups. Exploratory Cox proportional-hazards models included age, FIGO stage, and diagnosis era; post-baseline treatment completion and maintenance therapies were not included as fixed covariates because of potential guarantee-time bias and incomplete exposure dates. **Results**: The cohort comprised 44 patients aged 70–74 years, 38 aged 75–79 years, and 15 aged 80 years or older. Recorded treatment groups were primary debulking surgery (PDS; *n* = 46), NACT followed by recorded interval debulking surgery (NACT + IDS; *n* = 22), chemotherapy alone (*n* = 22), and no treatment (*n* = 7). Among the 90 patients in the PFS population, 61 experienced an event; among all 97 patients, 36 died. Median PFS was 22.0 months (95% CI, 16.0–24.0) and median OS was 51.5 months (95% CI, 38.0–not reached). Age-group log-rank *p* values were 0.684 for PFS and 0.248 for OS. The baseline Cox models were exploratory and showed no statistically significant association between age and PFS or OS after adjustment for FIGO stage and diagnosis era. **Conclusions**: This retrospective cohort does not establish that age, surgery, treatment completion, or maintenance therapy causes a survival difference. Survival estimates among patients aged 80 years or older were imprecise. Treatment comparisons should be interpreted as selected-cohort associations, and prospective studies incorporating validated geriatric, comorbidity, molecular, and time-dependent treatment information are needed.

## 1. Introduction

Epithelial ovarian cancer (EOC) is a major cause of gynecologic cancer mortality and is frequently diagnosed after dissemination beyond the pelvis. Older adults represent a clinically heterogeneous population in whom chronological age may coexist with frailty, comorbidity, functional impairment, nutritional vulnerability, and altered pharmacologic tolerance. These factors can influence both the feasibility of surgery and systemic therapy and the interpretation of observational survival comparisons [1,2,3,4].

Contemporary treatment decisions for advanced EOC commonly consider primary debulking surgery (PDS) versus neoadjuvant chemotherapy (NACT) followed by interval debulking surgery (IDS), together with patient fitness, disease distribution, and the likelihood of achieving meaningful cytoreduction. Current Society of Gynecologic Oncology/American Society of Clinical Oncology guidance and randomized trials support individualized selection between these strategies rather than an age-only approach [5,6,7,8].

Maintenance treatment with bevacizumab or a poly (ADP-ribose) polymerase (PARP) inhibitor has become part of modern ovarian-cancer care for selected patients [9,10,11,12]. However, eligibility, molecular testing, access, treatment initiation, duration, and survival to treatment are not exchangeable across patients. Therefore, a retrospective comparison of maintenance-treated and untreated patients should not be interpreted as a treatment-efficacy analysis without appropriate time-dependent or landmark methods [13].

Evidence specifically focused on patients aged 80 years or older remains limited. The present study was designed to describe treatment patterns and survival associations rather than to estimate causal treatment effects [4,14,15].

The primary research question was: among patients aged 70 years or older with recorded FIGO stage IIB–IV EOC-spectrum disease, how do age-stratified survival outcomes and recorded treatment patterns compare? The prespecified primary outcomes were PFS and OS. Treatment-pattern distributions and operative morbidity were considered secondary or exploratory outcomes.

## 2. Methods

### 2.1. Study Design and Population

This retrospective cohort study used institutional clinical records for patients aged 70 years or older who were identified between January 2010 and December 2023. The inclusion of FIGO stage IIB was retained because it was part of the institutional study cohort; stage IIB was reported separately and was not combined with stage III–IV in the main descriptive tables [16]. Reporting was guided by the Strengthening the Reporting of Observational Studies in Epidemiology (STROBE) recommendations [17]. The institutional database initially contained 122 patients with suspected ovarian malignancy. The final study population included 97 patients with recorded FIGO stage IIB–IV disease after exclusion of incomplete records (*n* = 2), borderline tumors (*n* = 2), incorrect histology (*n* = 6), non-ovarian primary malignancy (*n* = 5), and early-stage disease (*n* = 10). All included cases had pathological confirmation based on biopsy or surgical specimens according to institutional pathology records. The cohort comprised ovarian, fallopian-tube, and primary peritoneal carcinomas under the study’s EOC-spectrum definition.

The primary-site distribution was 87 ovarian, 6 fallopian-tube, and 4 primary peritoneal carcinomas (97 total). No a priori sample-size calculation was performed; the cohort consisted of all eligible records in the institutional database. The database was locked on 15 January 2024.

### 2.2. Diagnosis and Treatment

All included cases had histopathological confirmation based on biopsy or surgical specimens. Treatment assignment was made by a multidisciplinary team using clinical condition, comorbidity, disease distribution, and perceived resectability. Formal geriatric assessment and standardized frailty measures were not consistently recorded, particularly before 2020. Performance status, comorbidity burden, renal function, quantitative tumor burden, and operability scores were not systematically recorded and therefore could not be reliably adjusted for [14,15].

Patients were classified according to the recorded treatment sequence as PDS, defined as primary debulking surgery with or without subsequent chemotherapy; NACT + IDS, defined as neoadjuvant chemotherapy followed by recorded interval debulking surgery; chemotherapy alone; or no treatment. Standard platinum-based chemotherapy was generally paclitaxel plus carboplatin, but regimen intensity and intraperitoneal chemotherapy varied over time. Maintenance treatment fields for bevacizumab and PARP inhibitors were incomplete and did not consistently capture exposure details. Because treatment initiation dates, treatment duration, molecular eligibility, and exact exposure status were not systematically recorded, maintenance therapies were not modeled as causal exposures in the primary analyses.

### 2.3. Variables and Outcomes

Variables assessed included age, presenting symptom, body mass index (BMI), baseline albumin, baseline CA-125, ascites grade, FIGO stage, histology, recorded tumor differentiation, treatment category, chemotherapy cycles, residual disease, Clavien–Dindo grade, diagnosis date, and recorded PFS and OS times. Histologic subtype and recorded differentiation were kept as separate variables.

PFS was defined as time from diagnosis to documented progression or death. Patients without progression or death were censored at the last documented follow-up. OS was defined as time from diagnosis to death, with survivors and patients lost to follow-up censored at their last recorded survival time. The PFS event definition counted the categories “progression,” “death,” and “death/without documented progression” as events; categories indicating no progression or loss to follow-up without an event were censored. Seven patients classified as receiving no treatment were excluded from the PFS population because PFS was defined among patients who entered treatment observation, whereas all 97 patients were included in the OS population.

Follow-up time was measured from the date of diagnosis to the date of death or the last documented contact, as applicable. Follow-up information was obtained through clinic visits, telephone contact, and medical-record linkage.

### 2.4. Statistical Analysis

Categorical variables are presented as counts and percentages; continuous variables are presented as medians and ranges or interquartile ranges. Age-stratified survival was estimated using the Kaplan–Meier method. Greenwood variance with a log–log confidence band was used for survival estimates, and median confidence intervals were obtained by inversion of the pointwise survival band. Multi-group log-rank tests were used to compare survival across age groups. All analyses were performed using the study dataset; no values were imputed [18].

Because treatment completion, IDS completion, PARP inhibitor use, and bevacizumab use are determined after diagnosis and can require survival and continued eligibility, these variables were not entered as fixed covariates in the primary Cox analysis. Instead, exploratory baseline Cox models included age as a continuous variable, FIGO stage (III and IV versus IIB), and diagnosis era (2016–2020 and 2021–2023 versus 2010–2015). The PFS model included 90 patients and 61 events with five parameters (12.2 events per parameter); the OS model included 97 patients and 36 deaths with five parameters (7.2 events per parameter). No propensity-score or inverse-probability analysis was performed because key treatment-selection covariates were not consistently recorded or were incompletely documented [13,19].

Age was modeled linearly. Exploratory model diagnostics included Schoenfeld-residual correlations with log time for each covariate, a likelihood-ratio comparison of linear versus quadratic age terms, and variance-inflation factors (VIFs). No material evidence of nonproportional hazards was observed in either model (all permutation-based *p* values > 0.15); adding a quadratic age term did not improve model fit (*p* = 0.621 for PFS and *p* = 0.577 for OS), and VIFs ranged from 1.04 to 2.89. Given the limited number of OS events (36 deaths), these exploratory diagnostics should be interpreted cautiously. All reported *p* values are two-sided and are reported as *p* < 0.001 when the exact value is below 0.001.

## 3. Results

### 3.1. Patient Flow and Baseline Characteristics

Of 122 screened records, 25 were excluded, leaving 97 included patients. The age distribution was 44 patients aged 70–74 years (45.4%), 38 aged 75–79 years (39.2%), and 15 aged 80 years or older (15.5%). The median age was 75 years (range, 70–93). The recorded FIGO distribution was IIB (*n* = 8), IIIB (*n* = 2), IIIC (*n* = 62), IVA (*n* = 6), and IVB (*n* = 19). Thus, 89/97 patients (91.8%) had stage III–IV disease; the eight stage IIB patients were retained because the cohort definition was FIGO IIB–IV.

**Figure 1:**
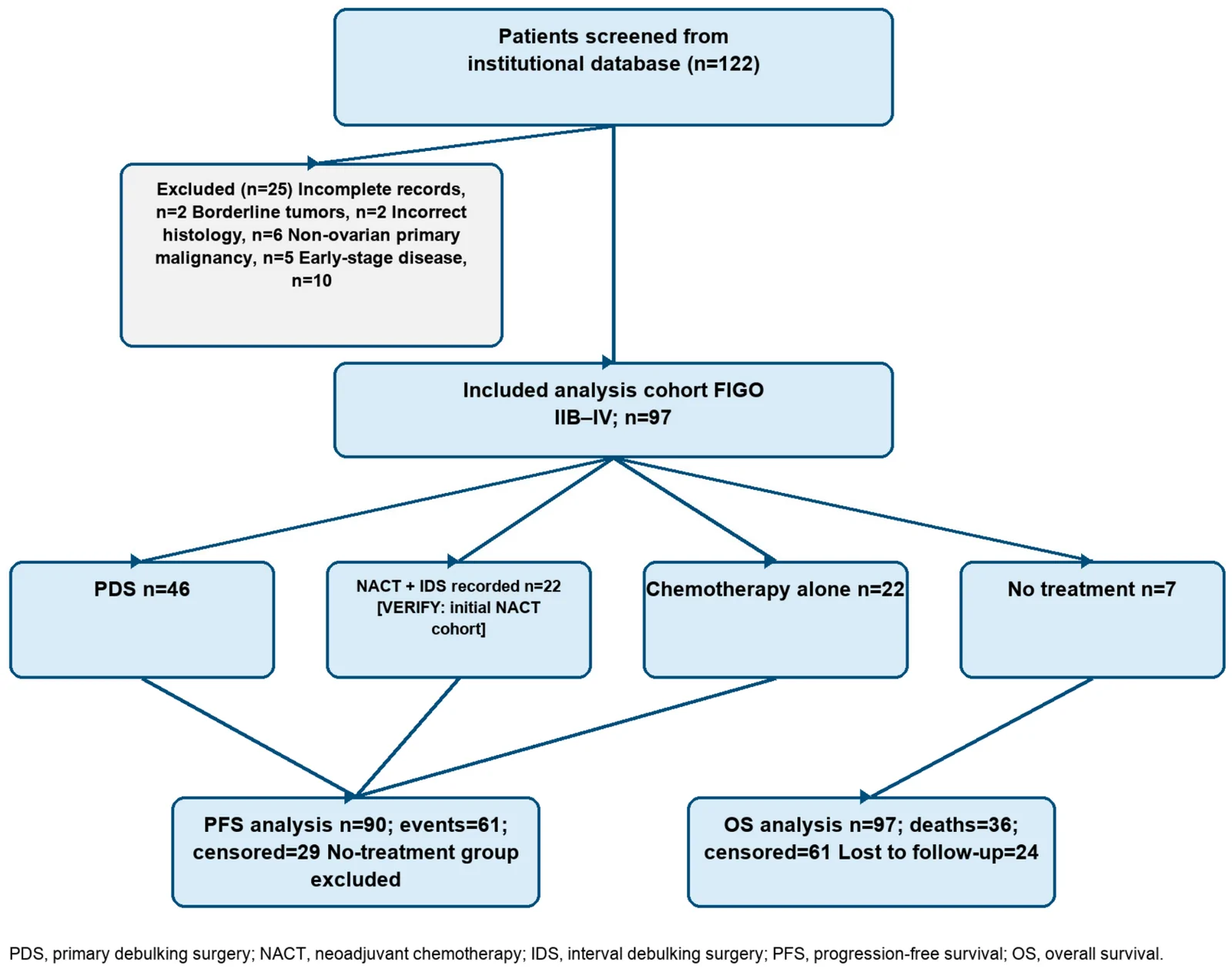
Participant flow and analysis populations. PFS excluded seven patients recorded as receiving no treatment; OS included all 97 patients. Treatment categories were defined from the recorded treatment-sequence field; 22 patients were classified as NACT + IDS.

The pathological profiles of the patients were as follows: serous carcinoma accounted for 79 patients, clear-cell carcinoma for 6, mucinous carcinoma for 3, carcinosarcoma for 3, mixed carcinoma for 3, endometrioid carcinoma for 2, and neuroendocrine carcinoma for 1. All included cases had pathological confirmation (Table 1 and Figure 1).

### 3.2. Treatment Patterns

Recorded treatment categories were PDS (*n* = 46, 47.4%; PDS alone, *n* = 8; PDS plus chemotherapy, *n* = 38), NACT + IDS (*n* = 22, 22.7%), chemotherapy alone (*n* = 22, 22.7%), and no treatment (*n* = 7, 7.2%). Treatment selection differed across age groups descriptively, with no untreated patients in the 70–74-year group, four in the 75–79-year group, and three in the group aged 80 years or older. These differences are not interpreted as causal effects of age.

### 3.3. Operative Outcomes and Corrected Complication Coding

Sixty-eight patients underwent recorded cytoreductive surgery (PDS, *n* = 46; IDS, *n* = 22). In the Clavien–Dindo classification [20], 62/68 patients (91.2%) had no recorded postoperative complication, four (5.9%) had grade II complications, and two (2.9%) had grade III complications; no grade I, IV, or V complications were recorded. The exact Fisher–Freeman–Halton test comparing the six-category distributions between PDS and IDS yielded *p* = 0.367 (Table 2).

### 3.4. Age-Stratified Survival

The corrected PFS population included 90 patients (61 events, 29 censored): 44 patients aged 70–74 years (30 events), 34 aged 75–79 years (22 events), and 12 aged 80 years or older (9 events). The OS population included all 97 patients, with 36 deaths and 61 censored observations; 24 patients were recorded as lost to OS follow-up, and these were a subset of the censored observations.

**Figure 2:**
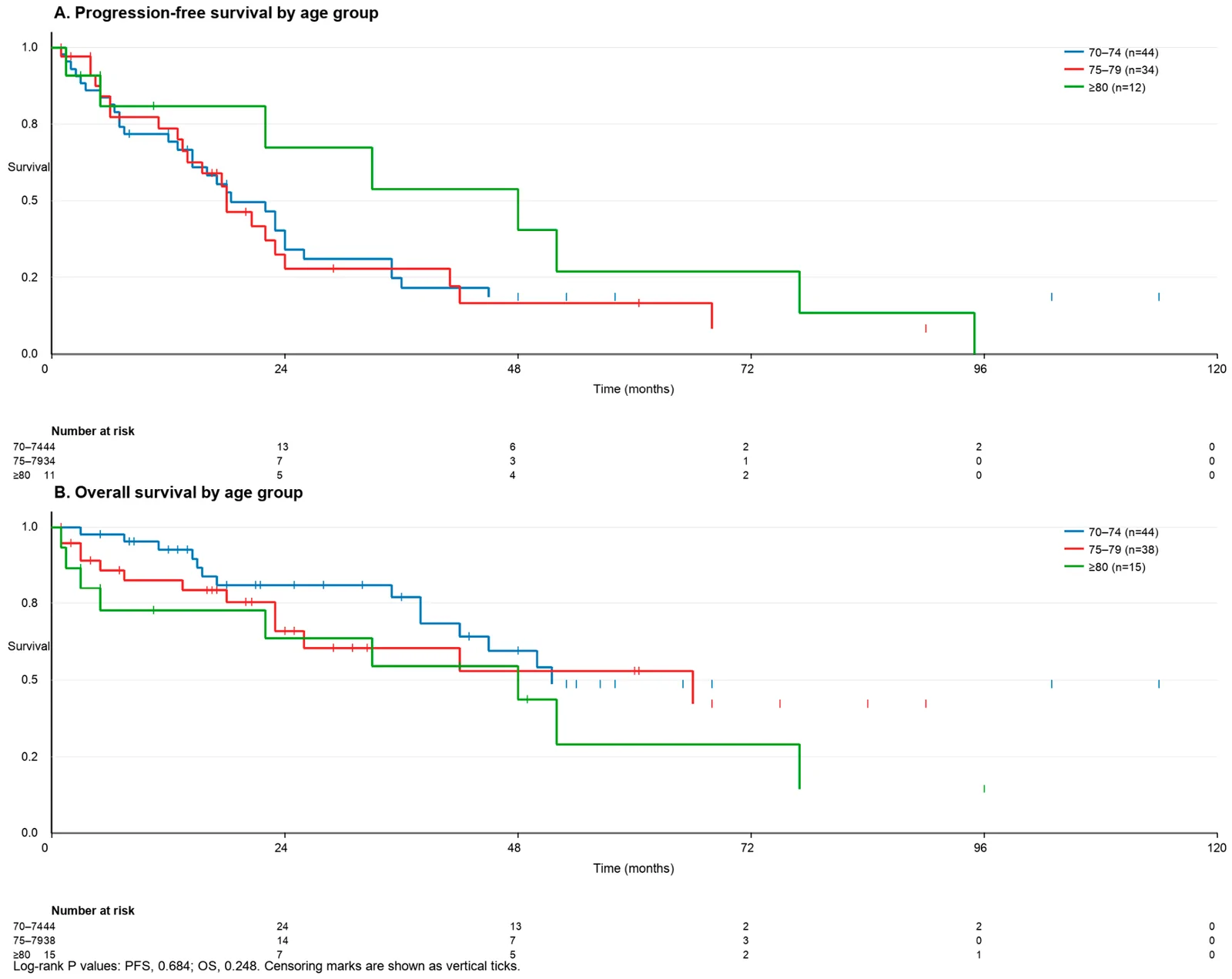
Kaplan–Meier estimates by age group. (**A**) PFS; (**B**) OS. Numbers at risk, censoring marks, and overall log-rank *p* values are shown.

Median PFS was 18.5 months (95% CI, 14.5–24.0) in patients aged 70–74 years, 18.0 months (95% CI, 13.5–24.0) in those aged 75–79 years, and 48.0 months (95% CI, 5.0–77.0) in those aged 80 years or older. Median OS was 51.5 months (95% CI, 38.0–not reached), 66.0 months (95% CI, 23.0–not reached), and 48.0 months (95% CI, 3.0–77.0), respectively. Overall log-rank tests did not show a statistically significant difference across age groups (PFS, *p* = 0.684; OS, *p* = 0.248). These *p* values do not demonstrate equivalence, particularly for the small ≥80-year group (Figure 2).

### 3.5. Exploratory Treatment-Group Survival Associations

**Figure 3:**
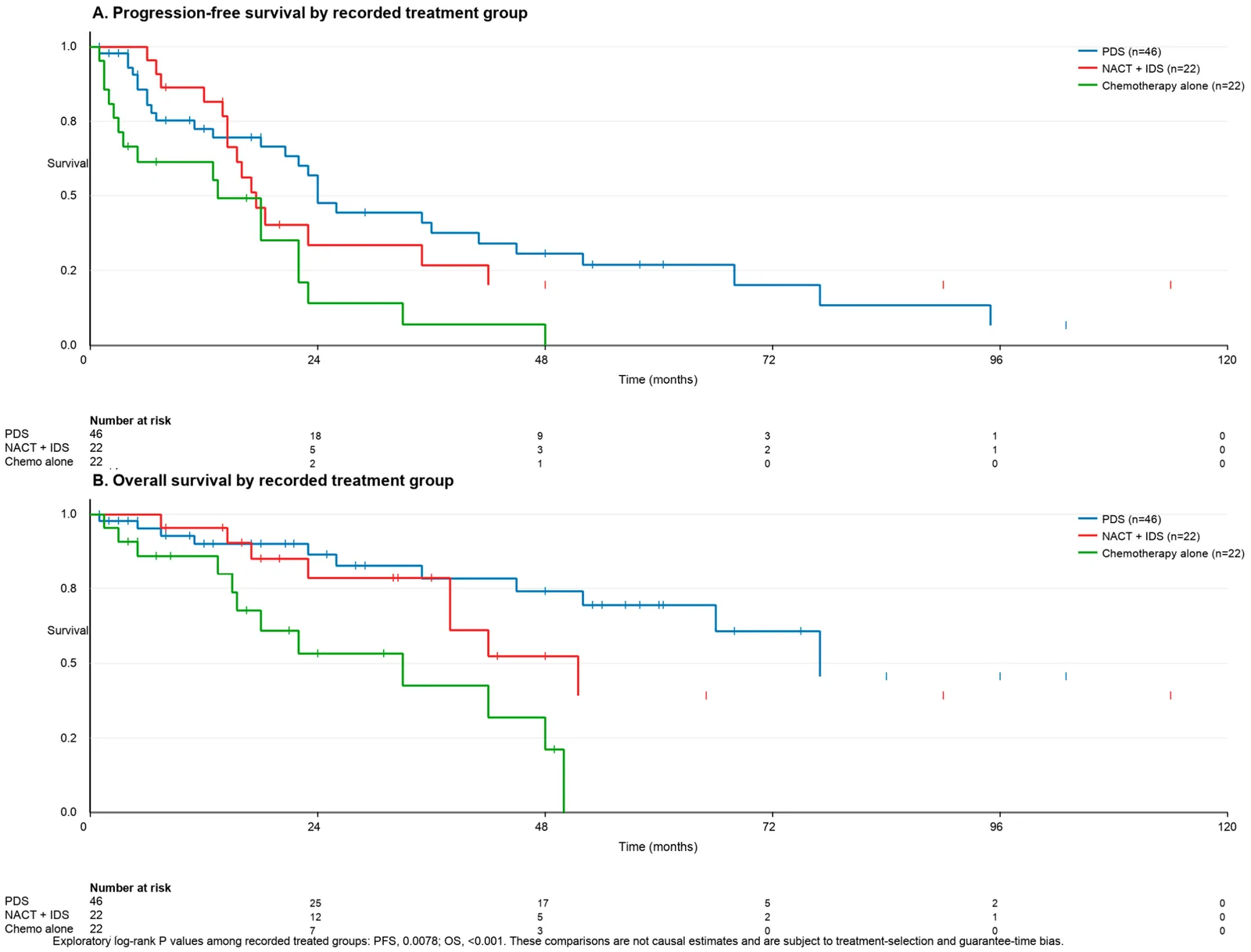
Exploratory Kaplan–Meier estimates by recorded treatment group. (**A**) PFS; (**B**) OS. These curves compare selected, non-exchangeable groups and are not causal treatment-effect estimates.

Among recorded treated groups, exploratory Kaplan–Meier estimates showed median PFS of 24.0 months for PDS (*n* = 46), 17.5 months for NACT + IDS (*n* = 22), and 13.5 months for chemotherapy alone (*n* = 22); the overall log-rank *p* value was 0.0078. Median OS was 77.0, 51.5, and 33.0 months, respectively; the overall log-rank *p* value was <0.001. These comparisons are descriptive associations among non-exchangeable groups and are vulnerable to confounding by indication, selection bias, and guarantee-time bias. They should not be interpreted as evidence that one treatment strategy causes longer survival (Figure 3).

### 3.6. Exploratory Baseline Cox Models

The primary Cox analysis excluded post-baseline treatment completion, IDS completion, PARP inhibitor use, and bevacizumab use from fixed-covariate models. The exploratory baseline models included age, FIGO stage, and diagnosis era. Age was not statistically associated with PFS (HR 0.976, 95% CI 0.918–1.038; *p* = 0.444) or OS (HR 1.036, 95% CI 0.973–1.104; *p* = 0.268) after adjustment for stage and era. Stage estimates were imprecise because only eight patients had stage IIB disease and only 36 OS deaths occurred (Table 3). Additional baseline, treatment, and outcome summaries are provided in the Supplementary Materials (Table S1–S4).

## 4. Discussion

This retrospective cohort study describes treatment patterns, operative morbidity, and survival in 97 patients aged 70 years or older with recorded FIGO stage IIB–IV EOC-spectrum disease. The main findings are that the cohort was heterogeneous, and the recorded treatment groups exhibited different survival distributions but were not clinically exchangeable. The study therefore supports descriptive reporting of real-world care rather than causal claims about treatment benefit.

The absence of a statistically significant age-group difference should not be interpreted as proof that chronological age has no prognostic relevance. The ≥80-year group contained only 15 patients overall and 12 in the PFS population, and its confidence intervals were wide. The findings may reflect selective inclusion of patients who reached the tertiary center and received treatment, as well as unmeasured differences in frailty, functional status, comorbidity, disease burden, and treatment eligibility.

The exploratory treatment curves showed longer survival among patients recorded as undergoing PDS than among those recorded as receiving NACT + IDS or chemotherapy alone. However, patients receiving these approaches were selected on the basis of clinical condition, disease distribution, resectability, clinician judgment, and subsequent survival. In addition, IDS completion and maintenance therapy occur after diagnosis and can create guarantee-time (immortal-time) bias when analyzed as fixed exposures [13]. The present data do not support the conclusion that PDS, treatment completion, bevacizumab, or PARP inhibitors caused a survival advantage.

The corrected operative morbidity data suggest that surgery was feasible in selected older patients, with 91.2% of operated patients having no recorded postoperative complication and no grade IV or V events. This observation is relevant to feasibility, but it does not establish a survival benefit from maximal cytoreduction or justify extrapolation to patients who were not considered operable.

Clinical interpretation should prioritize multidimensional assessment rather than chronological age alone. This is a clinical implication supported by geriatric-oncology guidance and prior studies, not a direct finding of the present cohort [14,15].

### 4.1. Limitations

This study has important limitations. It was retrospective, single-center, and conducted over 2010–2023, a period during which surgical selection, FIGO staging practice, systemic therapy, molecular testing, and maintenance-treatment availability changed. Calendar era was included in the exploratory baseline Cox models, but residual calendar-time bias remains possible. The dataset lacked structured ECOG performance status, ASA class, Charlson Comorbidity Index, formal frailty or geriatric assessment, renal function, quantitative tumor burden, detailed resectability assessment, and complete BRCA/HRD results. Therefore, confounding by indication and residual confounding are likely.

### 4.2. Strengths

Strengths include inclusion of patients aged 70 years or older, explicit separation of PFS and OS analysis populations, correction of the PFS event definition, correction of the Clavien–Dindo coding error, use of age-stratified log-rank tests, and transparent separation of descriptive treatment associations from causal treatment claims.

## 5. Conclusions

In this retrospective cohort of selected patients aged 70 years or older with recorded FIGO stage IIB–IV EOC-spectrum disease, age-group survival estimates did not show a statistically significant difference, but the study was not powered to demonstrate equivalence. Recorded treatment groups had different survival distributions, yet the differences are subject to confounding by indication and guarantee-time bias. The data support individualized assessment of fitness, frailty, comorbidity, disease burden, and treatment eligibility rather than treatment decisions based on chronological age alone; they do not establish a causal benefit of surgery, treatment completion, or maintenance therapy.

## Figures and Tables

**Table 1:** Baseline characteristics by age group.

Characteristic	70–74 Years (*n* = 44)	75–79 Years (*n* = 38)	≥80 Years (*n* = 15)	Overall (*n* = 97)
Age, years; median (range)	74.0 (70–74)	77.0 (75–79)	84.0 (80–93)	75.0 (70–93)
PDS	21 (47.7%)	18 (47.4%)	7 (46.7%)	46 (47.4%)
NACT + IDS	15 (34.1%)	7 (18.4%)	0 (0.0%)	22 (22.7%)
Chemotherapy alone	8 (18.2%)	9 (23.7%)	5 (33.3%)	22 (22.7%)
No treatment	0 (0.0%)	4 (10.5%)	3 (20.0%)	7 (7.2%)
FIGO IIB	4 (9.1%)	3 (7.9%)	1 (6.7%)	8 (8.2%)
FIGO III	32 (72.7%)	23 (60.5%)	9 (60.0%)	64 (66.0%)
FIGO IV	8 (18.2%)	12 (31.6%)	5 (33.3%)	25 (25.8%)
BMI, kg/m^2^; median (range)	23.4 (16.5–33.7)	24.5 (15.8–35.0)	25.0 (20.0–29.7)	24.1 (15.8–35.0)
Albumin; median (range)	37.7 (27.6–46.5)	28.9 (17.0–42.5)	32.5 (28.0–40.1)	34.8 (17.0–46.5)
CA-125; median (range)	649.0 (13.2–9439.0)	683.4 (13.1–6653.2)	1893.2 (16.3–13,509.0)	746.4 (13.1–13,509.0)
Chemotherapy cycles; median (range)	6.0 (0.0–11.0)	4.5 (0.0–13.0)	1.0 (0.0–6.0)	5.0 (0.0–13.0)
Ascites grade 1	18 (40.9%)	7 (18.4%)	3 (20.0%)	28 (28.9%)
Ascites grade 2	6 (13.6%)	7 (18.4%)	3 (20.0%)	16 (16.5%)
Ascites grade 3	20 (45.5%)	24 (63.2%)	9 (60.0%)	53 (54.6%)

Note: Values are descriptive. No formal baseline hypothesis tests were prespecified. PDS, primary debulking surgery; NACT, neoadjuvant chemotherapy; IDS, interval debulking surgery.

**Table 2:** Operative details and corrected postoperative complication classification.

Characteristic	Overall (*n* = 68)	PDS (*n* = 46)	IDS (*n* = 22)
Surgery performed	68 (100.0%)	46 (100.0%)	22 (100.0%)
Residual disease: no gross	29 (42.7%)	18 (39.1%)	11 (50.0%)
Residual disease: 1–10 mm	23 (33.8%)	19 (41.3%)	4 (18.2%)
Residual disease: >10 mm	16 (23.5%)	9 (19.6%)	7 (31.8%)
No postoperative complication	62 (91.2%)	41 (89.1%)	21 (95.5%)
Clavien–Dindo grade II	4 (5.9%)	4 (8.7%)	0 (0.0%)
Clavien–Dindo grade III	2 (2.9%)	1 (2.2%)	1 (4.5%)
Clavien–Dindo grade I, IV, or V	0 (0.0%)	0 (0.0%)	0 (0.0%)

Note: Operative details are descriptive. The postoperative-complication comparison used the exact Fisher–Freeman–Halton test across six mutually exclusive categories (no complication, grades I–V; *p* = 0.367).

**Table 3:** Exploratory baseline Cox models for PFS and OS.

Variable	PFS Model (*n* = 90; 61 Events)	OS Model (*n* = 97; 36 Deaths)
Age (per year)	0.976 (0.918–1.038); *p* = 0.444	1.036 (0.973–1.104); *p* = 0.268
FIGO stage III vs. IIB	6.198 (1.482–25.917); *p* = 0.012	4.852 (0.651–36.181); *p* = 0.123
FIGO stage IV vs. IIB	5.925 (1.313–26.730); *p* = 0.021	5.929 (0.741–47.413); *p* = 0.093
Diagnosis era 2016–2020 vs. 2010–2015	1.392 (0.743–2.608); *p* = 0.302	1.834 (0.831–4.049); *p* = 0.133
Diagnosis era 2021–2023 vs. 2010–2015	1.827 (0.834–3.999); *p* = 0.132	1.059 (0.303–3.702); *p* = 0.928

Note: HRs are from Breslow-ties exploratory Cox models with five parameters. PFS events per parameter = 12.2; OS events per parameter = 7.2. Age was modeled linearly. Exploratory Schoenfeld-residual, age-functional-form, and variance-inflation diagnostics did not identify material model violations. Given the limited number of OS events (36 deaths), these exploratory diagnostics should be interpreted cautiously. Treatment completion and maintenance-treatment variables were not modeled because their post-diagnosis timing and incomplete exposure data could introduce guarantee-time bias.

## Data Availability

The de-identified patient-level data are not publicly available because of institutional privacy restrictions but may be available from the corresponding author upon reasonable request after approval by the relevant authority.

## Supplementary Materials

The following supporting information can be downloaded at: https://www.mdpi.com/article/10.3390/jcm15207845/s1, Table S1: Baseline and treatment characteristics according to calendar era; Table S2: Recalculated Kaplan–Meier estimates by age group; Table S3: Analysis populations and outcome accounting; Table S4: Aggregate primary-site summary.
