# Supplementary material for: Treatment Patterns and Survival in Older Patients with FIGO Stage IIB–IV Epithelial Ovarian, Fallopian Tube, and Primary Peritoneal Carcinomas
Source: J Clin Med. 2026 Oct 12;15(20):7845. doi: 10.3390/jcm15207845 (PMC13705369; doi:10.3390/jcm15207845)
Supplement: Supplementary file 1 [file jcm-15-07845-s001.zip › jcm-4548566-supplementary.pdf]

Supplementary Tables for the Retrospective Cohort Study

These supplementary tables contain the recalculated analysis populations, calendar-era summaries, survival estimates, and primary-site summary.

Supplementary Table S1. Baseline and treatment characteristics according to calendar era

Characteristic	2010–2015 (n=26)	2016–2020 (n=42)	2021–2023 (n=29)
Age group, 70–74 years	11 (42.3%)	18 (42.9%)	15 (51.7%)
Age group, 75–79 years	13 (50.0%)	15 (35.7%)	10 (34.5%)
Age group, ≥80 years	2 (7.7%)	9 (21.4%)	4 (13.8%)
PDS + chemotherapy	9 (34.6%)	15 (35.7%)	14 (48.3%)
PDS alone	2 (7.7%)	6 (14.3%)	0 (0.0%)
NACT + IDS	7 (26.9%)	10 (23.8%)	5 (17.2%)
Chemotherapy alone	4 (15.4%)	8 (19.0%)	10 (34.5%)
No treatment	4 (15.4%)	3 (7.1%)	0 (0.0%)
Chemotherapy cycles, median (IQR)	6.0 (1.2–6.8)	4.0 (1.0–6.0)	5.0 (2.0–6.0)
No gross residual disease	10 (38.5%)	15 (35.7%)	4 (13.8%)
1–10 mm residual disease	4 (15.4%)	8 (19.0%)	12 (41.4%)
>10 mm residual disease	4 (15.4%)	8 (19.0%)	4 (13.8%)
Not operated	8 (30.8%)	11 (26.2%)	9 (31.0%)
Documented maintenance field nonblank	0/26 (0.0%)	9/42 (21.4%)	11/29 (37.9%)

Notes: Age-group distribution P=0.482; recorded primary-treatment distribution P=0.116; chemotherapy-cycle comparison P=0.543; residual-disease comparison P=0.183. PDS, primary debulking surgery; NACT, neoadjuvant chemotherapy; IDS, interval debulking surgery; IQR, interquartile range.

Supplementary Table S2. Recalculated Kaplan–Meier estimates by age group

Age group	PFS n	PFS events / censored	Median PFS months (95% CI)	OS n	OS deaths / censored	Median OS months (95% CI)
70–74	44	30 / 14	18.5 (14.5–24.0)	44	14 / 30	51.5 (38.0–NR)
75–79	34	22 / 12	18.0 (13.5–24.0)	38	13 / 25	66.0 (23.0–NR)
≥80	12	9 / 3	48.0 (5.0–77.0)	15	9 / 6	48.0 (3.0–77.0)

Overall multi-group log-rank P values were 0.684 for PFS and 0.248 for OS. Confidence intervals use Greenwood variance with a log–log survival band and inversion for the median; the ≥80-year estimates are imprecise.

Supplementary Table S3. Analysis populations and outcome accounting

Age group	PFS population	PFS events	PFS censored	OS population	Deaths	OS censored	Lost to OS follow-up
70–74 years	44	30	14	44	14	30	8 (18.2%)
75–79 years	34	22	12	38	13	25	13 (34.2%)
≥80 years	12	9	3	15	9	6	3 (20.0%)
Overall	90	61	29	97	36	61	24 (24.7%)

PFS excluded seven patients recorded as receiving no treatment; OS included all 97 patients. Lost-to-follow-up observations are a subset of censored OS observations and must not be added to the censored total. PFS events were progression or death; OS events were deaths.

Supplementary Table S4. Aggregate primary-site summary

Primary site	Patients, n (%)
Ovary	87 (89.7%)
Fallopian tube	6 (6.2%)
Primary peritoneum	4 (4.1%)
Total	97 (100.0%)
